# A quantum-cognitive approach to dynamic meaning construction

**DOI:** 10.3389/fpsyg.2026.1664747

**Published:** 2026-03-18

**Authors:** Meng Yin

**Affiliations:** 1 School of Philosophy and Social Development, Huaqiao University, Xiamen, China; 2 School of Foreign Languages, Huangshan University, Huangshan City, China

**Keywords:** dynamic meaning construction, large language models, metaphor comprehension, neural oscillations, photonic computing, quantum cognition, quantum semantics, Quantum-Native NLP

## 1 Introduction

Language isn't just a rigid system of symbols. Instead, it's a living, embodied phenomenon, deeply intertwined with our physical experience and shaped by our interaction with the environment (Wang, 2019; Zhou and Luo, 2024). However, the dynamic nature of language brings a significant challenge to cognitive science: the well-known “stability-plasticity dilemma” (Grossberg, 1980). On one hand, for clear communication, meanings of words need to be stable and widely recognized, so everyone can understand them, no matter when or who speaks them. On the other hand, these meanings must also be flexible and adaptable in varying contexts. While traditional computational models, from early generative grammar to standard Bayesian approaches, have excelled at modeling these stable meanings, they often treat semantic ambiguity as “noise” that needs to be eliminated, rather than a valuable resource (Gärdenfors, 2014).

Even with the significant “probabilistic turn” in cognitive science, which brought Bayesian models to handle uncertainty, most of these models still rely on classical probability theory. They assume the meaning of a concept is a pre-defined distribution over a set of fixed features. As Bruza and Cole (2005) pointed out, this dependence on classical set theory creates a major epistemological barrier because it treats semantic ambiguity as “noise” rather than a fundamental part of meaning construction. Though scholars have recently developed more complex tools, like Gradient Symbolic Representations (GSR), to model meanings as weighted mixtures (Smolensky et al., 2014; Mondal, 2024), these approaches are still limited by Kolmogorovian probability. They still follow the Law of Total Probability, which forces conflicting meanings to be simply added together and mixed. We argue that this basic “mixture” method isn't enough to describe or handle complex situations where meanings are incompatible or interfere with each other in context. Therefore, much empirical evidence suggests that capturing these dynamic features requires a non-classical, quantum probability framework (Surov et al., 2021).

The importance of this paradigm shift becomes most clear when we analyze how everyday language works and how we interpret deep meanings in complex literary works. A classic example of such “semantic superposition” is the iconic “big fish” in Ernest Hemingway's The Old Man and the Sea.Within the novel's narrative structure, this phrase isn't a static label. Instead, it operates simultaneously on multiple, even mutually exclusive, semantic levels. Here, it serves as a biological marlin, a worthy adversary, and a transcendent symbol of life's ultimate tragedy. A classical probabilistic model fails to capture the dynamic tension that a reader feels, because it forces these meanings to compete for probability mass, implying only one can be dominant. In contrast, human reading suggests that meaning exists in a “superposition” state. It stays that way until a specific context makes it “collapse” into a concrete interpretation. Crucially, these overlapping meanings aren't simple probabilistic blends. They are coherent “quantum states” within a complex adaptive system.

This study proposes that Quantum Cognition offers the necessary mathematical formalism to resolve the “stability-plasticity dilemma”. This is supported by its proven success in solving decision-making paradoxes in psychology (Busemeyer and Bruza, 2012; Widdows et al., 2023; Huang et al., 2025). We introduce an integrated quantum theoretical model. In this model, the interaction between embodied experience and linguistic context is characterized as a genuine quantum interference phenomenon. This framework reinterprets the tension between stability and plasticity through the lens of Wave-Particle Duality. In our model, the “particle” corresponds to the stable, discrete symbols used for communication. The “wave” captures the fluid, context-sensitive potential that allows for creative interpretation.

Next, by employing the mathematical formalism of Hilbert space, we will mathematically demonstrate how semantic ambiguity can be maintained as a useful resource, rather than mere noise. This approach effectively overcomes the limitations inherent in traditional methods like static vectors and gradient symbolic mixtures. To ground these abstract formalizations, we focus on the “Big Fish” motif in Hemingway's The Old Man and the Sea. Through this case analysis, we will reveal how meaning dynamically evolves, similar to “state vector collapse”. Our study also extends to address the fundamental limitations of current Artificial Intelligence, particularly Large Language Models (LLMs). We argue that current LLMs, relying heavily on static statistical correlations, lack the “grounding” for true understanding. Therefore, we propose a pathway toward Quantum-Embodied AI and photonic intelligent systems by incorporating quantum-semantic principles. These systems could mimic the non-algorithmic fluidity of the human mind. Quantum probability is not an exotic addition to linguistics but a fundamental requirement for describing dynamic meaning. The research will first analyze the evolution from gradient representations to quantum interference, then formally express the wave-particle duality of meaning using mathematical methods. We will then validate this theoretical framework through the “big fish” case study and neurophysiological evidence, concluding with an exploration of its practical implications for Generative AI and Photonic Computing.

## 2 Evolution from static representations to quantum interference

To explain why we need the “quantum cognition” approach, we review the evolution of semantic theory. We will focus on why past mathematical models often failed to capture the dynamic, oscillating nature of meaning. We will examine how meaning representation started with static symbolic systems, then evolved to advanced gradient models. Next, we will identify the structural limitations of these older methods; these limitations force a paradigm shift toward a quantum probability framework. Indeed, this “quantum turn” in semantics has deep philosophical grounding. For example, Dalela (2012) argues that the basic structures of quantum theory, such as states, eigenstates, and measurement, can be systematically interpreted as structures of human meaning and interpretation. Thus, quantum theory itself can be seen as a framework for describing meaning.

### 2.1 The geometric rigidity of classical approaches

For a long time, attempts to formalize “meaning” were constrained by metaphors like “meaning is a container” or “meaning is a location”. In classical computational linguistics, especially Distributional Semantics, this metaphor is implemented through high-dimensional vector spaces. Models like Latent Semantic Analysis (LSA) and Word2Vec algorithms define a word's meaning by its fixed coordinates in a geometric space, calculated from co-occurrence statistics (Boleda, 2020). While this method effectively captures stable relationships, such as the vector analogy between “king” and “queen”, it becomes rigid when encountering word ambiguity.

Günther et al. (2019) point out that assigning a single, static vector to a polysemous word like “bank” (financial institution vs. riverbed) creates a “representational bottleneck”. This vector becomes a weighted average of all its meanings, placing the word in a semantic “no-man's-land” that is ambiguous to any single definition. Although advanced contextualized embedding models (like BERT or GPT architectures) try to resolve this with dynamic vectors, they are still fundamentally based on Classical (Kolmogorovian) Probability Theory. They treat “context” as a conditional variable that simply shifts the vector's position. However, they lack an “intrinsic mechanism” to model the word's ambiguous state before the context is fully processed (Haber and Poesio, 2023). Crucially, these models assume commutativity — that the order of information processing does not change the final result (A × B=B × A). This assumption is contradicted by psychological research on “order effects”, which show that the sequence of information fundamentally alters judgments and interpretation (Müller et al., 2017; Stricker et al., 2020).

Besides these computational methods, Cognitive Grammar offers profound insights into how meaning arises from “embodied experience” (Langacker, 2008). Scholars like Geeraerts (2009) have stated that meaning is not a fixed point but a dynamic construal. However, Cognitive Linguistics has historically lacked formal mathematical tools to describe the simultaneous, probabilistic activation of mutually exclusive meanings. Similarly, Conceptual Blending Theory (Fauconnier and Turner, 2002), while enhancing our understanding of metaphor, remains largely descriptive. It can explain how concepts connect, but its predictive power is limited regarding how these concepts probabilistically “collapse” in real-time processing (Shain et al., 2024; Atanasov et al., 2025).

### 2.2 Gradient symbolic representations

To address the shortcomings of static vectors, a major step forward is the “Gradient Symbolic Representations (GSR)” theory, introduced by Smolensky et al. (2014). We can think of GSR as a sophisticated bridge that tries to connect the fluid, continuous changes of neural networks with the clear, structural demands of symbolic logic. Unlike traditional vector models that either fully activate a symbol or not at all (binary, all-or-nothing), GSR allows a symbol to have “partial activity”. This approach (formalism) creates a computational state where several grammatical or semantic meanings can exist simultaneously as a weighted mixture. This provides a more detailed picture of how our brains process language (Hsu, 2022).

Building on GSR, Mondal (2024) recently improved this method. He cleverly used gradient symbols to combine the “rigor” of logic with the fluidity typical of cognitive semantics. His research shows that linguistic meaning is better modeled as a continuous trajectory, not a discrete step function. This allows for “fuzzy” membership in semantic categories, aligning with recent evidence that word meaning is both categorical and continuous (Trott and Bergen, 2023). By recognizing the graded nature of human categorization, this model significantly improves upon binary systems. It captures the subtle idea that a concept can partially belong to several categories at once.

However, a crucial theoretical limitation still exists. Despite these advancements, both the original GSR framework and Mondal's new model strictly follow the basic rules of classical probability. In these gradient models, multiple meanings coexisting are seen as an “epistemic mixture”, not a true “ontic superposition”. This limitation has led researchers in cognitive science to explore quantum probability alternatives (Pothos and Busemeyer, 2022; Blutner and Graben, 2016). This distinction is very important, and we can explain it with an example: a classical gradient model views an ambiguous word like a coin that has already landed on heads or tails, but is covered by a hand. Here, our uncertainty (observer's) is simply a lack of knowledge. In contrast, quantum probability suggests its state is more like a spinning coin, which is simultaneously heads and tails, a dynamic state until context “stops” or “measures” it.

Mathematically, this limitation shows up in their strict adherence to the Law of Total Probability. This law states that the probability of a combined event is just the simple sum of its separate parts. We argue that this “mixture” model is insufficient for analyzing complex metaphors or polysemy. These linguistic phenomena are characterized not just by blending concepts, but by interference.

This theoretical argument is supported by experimental evidence. Surov et al. (2021) studied “quantum semantics” in text perception. They found that human judgments of semantic similarity often violate classical probability rules (inequalities), especially the “Sure-Thing Principle” (Moreira and Wichert, 2016). Their research revealed that the interaction between a word and its context creates interference terms, similar to the constructive and destructive wave patterns in a double-slit experiment. These interference effects can non-linearly suppress some interpretations while enhancing others, a phenomenon impossible to model under classical theory. Therefore, this suggests that the human mind processes ambiguity not as a lack of information (a mixture), but as a useful, coherent cognitive resource (superposition).

### 2.3 The quantum turn in cognition and communication

Classical representations inherently have limitations, especially in set-theoretic and truth-conditional semantics. This has increasingly led to a “quantum turn” in semantics. This interdisciplinary shift aims to tackle challenges like graded membership, context-sensitive concept combination, and non-monotonic meaning shifts (where new information might change or even reduce initial meaning).

Aerts and his team did pioneering work, using quantum theory's “Hilbert space formalism” to represent concepts. This means a concept isn't static; it changes like a quantum state based on context and interaction. They successfully solved the famous “Pet-Fish problem” and similar issues, doing so with quantum phenomena like superposition and interference, instead of simple set intersection as before (Aerts and Czachor, 2004; Aerts and Gabora, 2005; Aerts, 2009). This opened a new direction for quantum formal semantics: “emergent meanings” in language appear as fresh quantum states, not as a simple combination of fixed features (compositional intersection), unlike building with blocks (Aerts et al., 2012). This approach fundamentally changed how we understand and process meaning, breaking rigid traditional thinking.

Beyond specific concept combinations, the broader framework of quantum cognitive science offers strong theoretical support for non-classical structures in natural language meaning. Atmanspacher (2017) clearly distinguished between “quantum brain” and “quantum mind” approaches. He stressed that mental processes can formally show features like quantum phenomena, such as complementarity, non-commutative operations, and non-Boolean logic. However, this doesn't necessarily mean that direct quantum physical effects happen in our brains. These “quantum-like characteristics” are believed to enable more flexible, context-dependent cognitive processing. In this processing, the order of our thoughts affects the outcome, and meaning might emerge with “unsharp truth values”, like a gray area, reflecting varying plausibility. Crucially, Atmanspacher directly discussed how these quantum principles apply to semantic networks and concept decomposition. Using “entanglement-style features” in quantum representations of concepts helps explain why the meaning of a combination isn't simply separable from its parts.

In computational linguistics, Hilbert-space-based semantic space models (like LSA-type vector spaces) were later found to be quantum-compatible. This means their geometric structure can accommodate quantum effects, such as contextual collapse of meaning and “non-monotonic update” of associations (where new information might change or overturn prior understanding, rather than just adding to it). Bruza and Cole (2005) explicitly proposed that semantic spaces can be seen as true “Hilbert spaces”. They deeply explored how quantum logic over subspaces can model context effects and our “deep-level” practical reasoning. In this way, they linked the dynamics of word meaning to generalized quantum logic.

Parallel work on the mental lexicon and word associations argued that associative networks display entanglement-like dependencies that are better captured by quantum probability than by classical spreading activation (Bruza, 2010). Experimental tests using conceptual combinations and sentence-like structures have now directly shown violations of Bell-type inequalities in semantic judgments, supporting the view that entanglement is a structural feature of meaning composition (Aerts et al., 2016, 2025).

Quantum-computational approaches to semantics (e.g., quantum computational logic; categorical/diagrammatic models) interpret sentence meanings as quantum information structures (qubit systems, density operators) and explore both compositional and holistic quantum semantics (Chiara et al., 2007). Category-theoretic work connects conceptual spaces with quantum concepts, showing how structured concepts can be learned and represented in quantum state spaces, with entanglement and quantum negation as specifically non-classical resources for modeling conceptual structure and semantic relations(Tull et al., 2024).

The “quantum turn” in semantics has profoundly impacted computational language technologies and the design of intelligent systems capable of human-like meaning processing. Self-organization algorithms based on “quantum-like principles” can make intelligent control systems more efficient (Litvintseva and Ulyanov, 2009). For example, “quantum-inspired language models” treat words as “superpositions over latent semantic units” and use “entanglement embeddings” to find “non-classical correlations” in word sequences. This improves performance on tasks like question answering and explains “semantic entanglement” (Chen et al., 2023). Meanwhile, “Quantum Natural Language Processing (QNLP)” goes further by directly mapping compositional distributional semantics to “quantum circuits”, using genuine quantum processes to build syntax-sensitive sentence meanings (Lorenz et al., 2021). This method allows AI to manage multiple potential interpretations or semantic states in superposition, which then dynamically “collapse” based on context, mirroring quantum measurement. This paradigm shift will move semantic analysis from rigid, rule-based methods to more flexible, context-sensitive models, which is crucial for AI to understand human-like, dynamic meaning.

The dynamic interaction between speaker and listener, especially when interpreting ambiguous or metaphorical utterances, can also be explained using quantum game-theoretic ideas. Eisert, Wilkens, and Lewenstein pioneered the concept of “quantum games”. Their theory shows that introducing “superposition” and “entanglement” into “strategic interactions” can turn classically “Pareto-inefficient dilemmas”, like the “Prisoner's Dilemma”, into cooperative, “Pareto-optimal equilibria” (Eisert et al., 1999). Similarly, quantum versions of the Monty Hall game illustrate how quantum strategies and measurements reshape information flow and optimal decision-making in counter-intuitive ways (Flitney and Abbott, 2002).

Previously, we analyzed the structural limitations of traditional semantic models, highlighting their inherent difficulty in fully capturing the graded, context-dependent, and critically, the interfering nature of linguistic meaning. This realization has greatly driven the “quantum turn” in cognition and communication research, laying an important conceptual foundation for understanding “non-classical cognitive processes” in semantics. While this fundamental shift has provided strong evidence and initial models for “quantum-like phenomena” in meaning representation, a clearer, more complete “theoretical framework” is still needed to precisely explain the dynamic changes and construction process of meaning within a comprehensive quantum-probabilistic paradigm. Building on these basic insights, we now propose a new theoretical framework based on an “ontological hypothesis”: the operations of our brain's Mental Lexicon are formally very similar to quantum mechanics. The core principle of this framework is the “Wave-Particle Duality of Meaning”, used to understand “semantic processing”, offering a robust explanation for the “dynamic oscillation” and “context-sensitive emergence” of meaning.

## 3 Theoretical framework: the wave-particle duality of meaning

This framework proposes an ontological hypothesis: the operations of our mental lexicon are formally very similar to quantum mechanics. It directly addresses the long-standing “stability-plasticity dilemma” in cognitive science. Language needs to be stable and precise to convey meaning, yet flexible enough to generate new communicative meanings based on context. We argue that this “duality” is functionally equivalent to the famous “Wave-Particle Duality” principle of quantum mechanics.

### 3.1 From static storage to dynamic superposition in psycholinguistics

Modern “psycholinguistic models” have abandoned the idea of the mind as a “dictionary”. Previously, finding a word was like looking up a static, discrete token in a dictionary. But now, increasing evidence supports a dynamic view where lexical organization and use are constantly changing. Libben's quantum-inspired morphological superposition theory rigorously elaborates on this, positing that lexical entries are more like potentials than fixed, stored words (Libben, 2017). Rodd's ambiguity-focused account aligns with this dynamic view, suggesting that accessing word meaning is like settling into a stable point within a “high-dimensional semantic space”, where multiple candidate meanings are simultaneously active and shaped by “contextual constraints” (Rodd, 2019).

This psychological reality mirrors the “superposition principle” of quantum mechanics. Based on the theoretical foundations by Busemeyer and Bruza (2012) and Aerts and Gabora (2005), we propose that a concept should not be seen as a fixed set of attributes (like a “particle”), but as a “state of potentiality” (like a “wave”). This state is represented by “complex probability amplitudes”, encompassing possibilities for various contextual outcomes (Khrennikov, 2015, 2020). In this model, a lexical item exhibits a dual ontic state depending on its processing phase. In the pre-activation phase, akin to a word resting in the mental lexicon, it behaves as a probability wave, representing an indefinite superposition of all possible senses. For instance, the uninstantiated word “bank” can be modeled as a “coherent superposition” of multiple semantic potentials, such as {|financial_ institution〉, |river_ bed〉, |tilt〉}, each corresponding to a context-selectable conceptual state (Aerts and Beltran, 2021). In this pre-activation “wave state”, the lexical item retains a reservoir of non-exclusive candidate meanings, effectively a “high-entropy superposition” with strong interference potential among alternative semantic components (Pothos and Busemeyer, 2022).

Conversely, when we use a word in a specific syntactic or pragmatic context, this act functions as a “Measurement Operator”. This interaction triggers a “State Vector Collapse”, where the diffuse wave function instantly becomes a discrete, definite “particle” of meaning, fitting perfectly into the specific communicative slot. This “duality” effectively explains why traditional models, which treat words solely as “particles”, consistently fail to capture the non-linear dynamic changes in meaning construction.

### 3.2 Mathematical formalism: Hilbert space semantics

To operationalize our ontological view of the essence of meaning, we employ the mathematical framework of Quantum Probability Theory (QPT), specifically “Complex Hilbert Space (H)”. Why this complex math? Because traditional classical set theory or “Euclidean vector spaces” (our everyday geometric spaces) have limitations. Especially, Kolmogorovian probability axioms fall short when dealing with contextuality and interference effects in meaning (Khrennikov, 2015).

#### 3.2.1 The superposition state and complex amplitudes

In our model, a word's meaning (semantic state) |ψ_*w*_〉 is considered as a unit vector in a complex Hilbert space H. The state is a linear combination of many independent, “pure” meaning basis vectors {|e_*j*_〉}, This “superposition” can be expressed by Equation 1:


$$|\psi_{w}\rangle =\sum_{j}\alpha_{j}|e_{j}\rangle$$ (1)

Most importantly, the coefficient α_*j*_ in the formula is not a real number (like classical neural network weights) but a “complex probability amplitude”. It consists of two parts: a magnitude *r*_*j*_ and a Phase Angle θ_*j*_, as defined in Equation 2:


$$\begin{array}{l} \alpha_{j}=r_{j}e^{i\theta_{j}} \end{array}$$ (2)

Here, i is the “imaginary unit” ($\sqrt{-1}$). The introduction of the phase angle θ is the core theoretical pillar of our model. In quantum-like cognition research, phase angles are not directly observable in single probability outcomes, but they subtly determine how a cognitive state will “interfere” with alternative states during measurements in different contexts. Geometrically, they describe the relationships between contexts on the “Bloch sphere” (Surov, 2021). So, from a cognitive perspective, this “phase dimension” can be understood as a “latent mental perspective” or “contextual resonance”. It's invisible in classical probabilistic descriptions but plays a decisive role in meaning's interactions and context-dependent state changes.

#### 3.2.2 The law of interference

The power of this formalism becomes evident when calculating the probability of a combined meaning. In Classical Probability Theory, the probability of the union of two events is simply the sum of their individual probabilities. However, in Quantum Probability, we apply the Born Rule to the sum of amplitudes, which generates an extra term. For a superposition involving two component states ψ_*A*_ and ψ_*B*_, the total probability P is:


$$\begin{array}{l} P\;=\;|\psi_{A}+\psi_{B}{|}^{2}=|\psi_{A}{|}^{2}+|\psi_{B}{|}^{2}+2|\psi_{A}||\psi_{B}|\cos\left(\theta_{A}-\theta_{B}\right) \end{array}$$ (3)

The term 2∣ψ_*A*_∣∣ψ_*B*_∣cos(θ_*A*_−θ_*B*_) is the Interference Term. When phases are aligned (cos(Δθ) > 0), the meaning is amplified (Constructive Interference), modeling the “resonance” felt in a powerful metaphor. Conversely, when phases are opposed (cos(Δθ) < 0), the meaning is suppressed (Destructive Interference). Equation (3) mathematically demonstrates why human semantic processing can violate classical logic (Surov et al., 2021).

#### 3.2.3 Contextual entanglement via tensor products

Language is inherently compositional, and a word never exists in a semantic vacuum. To model the interaction between a word (w) and its context (c), we adopt the categorical compositional framework (DisCoCat) proposed by Coecke et al. (2010). We describe the composite semantic space using the Tensor Product (⊗) in Equation 4:


$$\begin{array}{l} H_{total}\;=\;H_{w}\;⊗H_{c} \end{array}$$ (4)

Such tensor product constructions have been fruitfully used in compositional distributional semantics and quantum-inspired NLP to capture intra- and inter-modal interactions that are not reducible to independent word-level features (Coecke et al., 2013; Liu et al., 2023). Within this higher-dimensional space, the semantic state |ψ_*total*_〉 can become entangled. Unlike a simple separable state (product state), an entangled state implies that the semantic properties of the word and context are no longer independent; the state of the word is functionally dependent on the measurement of the context. This mirrors the philosophical idea of meaning holism: the meaning of a complex expression is an emergent property of the whole linguistic system and cannot be reduced to a simple sum of its parts (Andreas, 2010; Pradhan, 2019; Fabry et al., 2025).

#### 3.2.4 Time evolution of meaning

Meaning is not static; it changes as the narrative or sentences progress. We propose that the cognitive processing of a sentence involves a “unitary evolution”, controlled by a ”Semantic Hamiltonian (Ĥ)”. The “time-evolution” of the meaning state |ψ(*t*)〉 is shown by the Schrödinger equation (Equation 5):


$$i\hbar \frac{\partial }{\partial t}|\psi (t)\rangle =\hat{H}|\psi (t)\rangle$$ (5)

Similar unitary (or near-unitary) evolution equations have aslo been proposed to explain “order effects” and dynamic changes in cognitive states in sequential tasks (Busemeyer and Bruza, 2012; Khrennikov, 2015). Here the “Hamiltonian operator Ĥ ” represents the “energy landscape” or the syntactic tension of the sentence. This equation shows how the mental state “rotates” in the Hilbert space over time, modeling the “oscillation of meaning” a reader experiences before reaching a final interpretation.

## 4 Case study: quantum simulation of the fish metaphor in *The Old man and the Sea*

To validate the theoretical framework we discussed earlier, we're now moving from abstract mathematical formulas to a practical example. We'll use the “Hilbert space” approach to analyze the central metaphor of “fish” in Ernest Hemingway's famous novel, The Old Man and the Sea (2016). Our specific goal is to “quantify” how words interact in a “conflict context” and show how meaning collapses into a definite understanding. This “quantum simulation” isn't just for illustration; it's a rigorous “proof-of-concept”. It will demonstrate how “quantum probability amplitudes” can mathematically model the emergence of deep meaning from “contradictory semantic cues”, a task that classical logic-probabilistic models have consistently struggled with when dealing with “paradoxical conceptual combinations” (Wang et al., 2013; Pothos and Busemeyer, 2022).

### 4.1 The paradox of the signifier

In Hemingway's novel, the lexical item “fish” acts as a “dual signifier”, simultaneously representing two distinct “ontological planes”. On one hand, the novel vividly describes the fish's biological reality, with purple stripes, a huge tail, and the smell of blood and salt. This shows its biological aspect. At the same time, the story elevates it to a spiritual symbol; the protagonist Santiago calls the fish his “brother”, a noble thing, and an equal in their fight for dignity. This “tension” peaks in Santiago's pivotal statement:

“Fish,” he said, “I love you and respect you very much. But I will kill you dead before this day ends.” (Hemingway, 2016: 60)

In a traditional semantic network (like WordNet or standard Vector Space Models), the concepts of “Brother/Love” and “Prey/Kill” are usually opposing. They exist in distant, negatively correlated regions of a high-dimensional space. From a cognitive perspective, processing such conflicting concepts simultaneously would typically lead to high “cognitive load” and dissonance. Therefore, a classical logic model would predict that thinking about “Kill” would suppress the idea of “Love”, leading to semantic cancellation or ambiguity. However, the reader's phenomenological experience isn't one of contradiction or confusion, but a heightened, resonant truth. This suggests that the human mind doesn't perform simple logical subtraction (Love – Kill = Confusion); instead, it engages in non-linear integration, skillfully blending physical reality with spiritual abstraction. Our aim is to mathematically simulate this amplification of meaning.

### 4.2 State initialization

To mathematically represent the “semantic duality” mentioned above, we define a “two-dimensional complex Hilbert space H”. This space is spanned by two “orthonormal basis vectors”, each representing a different “ontological plane” in the novel. In this framework, the “basis vector |B〉” stands for the “Biological eigenstate”, meaning the fish is purely an animal, food, and a creature of instinct. The other “orthogonal vector |S〉” represents the “Spiritual eigenstate”, portraying the fish as a noble adversary, a “brother”, and a symbol of nature's tragic dignity.

When the story hasn't unfolded yet, or when the lexical item “fish” is encountered in isolation, its meaning is uncertain; it's in a state of “maximum entropy”. To capture the symmetry between its biological and spiritual interpretations, we assume a “symmetry-based indifference principle”. This means we assign equal prior weights to both the biological eigenstate and the spiritual eigenstate, representing “fish” in a “balanced superposition state”. Thus, the “state vector |ψ_*fish*_〉” is constructed as a coherent linear combination in Equation 6:


$$|\psi_{fish}\rangle =\;\frac{1}{\sqrt{2}}|\;B\rangle +\;\frac{1}{\sqrt{2}}|S\rangle \approx \;0.707|B\rangle +\;0.707|S\rangle$$ (6)

This initialization implies a fundamental “mathematical symmetry”. Before a specific narrative context forces “semantic collapse”, the probability of accessing either the biological or spiritual meaning is identical ( |0.707|^2^ = 0.5). Crucially, the “phase difference” between the two components is initially set to zero, representing a coherent potentiality where both interpretations coexist without interference. This mathematical representation simulates a superposition-like cognitive state, where the system holds multiple potential interpretations in active suspension until a specific context triggers collapse to a single, contextually appropriate meaning (Fuyama, 2023).

### 4.3 Deriving the conflict context operator

The core of our analysis is how the specific narrative context of the novel forces the previously uncertain meaning of “fish” to become definite (“collapse”). Unlike traditional models that treat context as a static filter, merely removing information, our “quantum model” defines narrative context as a Rotation of the Measurement Basis”. This reflects the cognitive act of shifting one's perspective. Hemingway's unique stylistic achievement lies in creating what we call “Grounded Spirituality”, where transcendence is achieved through physical struggle rather than by abandoning it.

To quantify this perspective shift, we introduce a “Context Vector” (|C_*conflict*_〉) that rotates the reader's cognitive focus. This rotation angle φ is non-arbitrary. It represents the text's precise “semantic orientation”. We believe that extreme angles fail to capture the “literary nuance”: for example, a rotation of 0° (aligning purely with the |S〉 axis) would make the novel an abstract allegory lacking realism; while a rotation of 90° (aligning purely with the |B〉 axis) would resemble a dry biological report. And a median angle of 45°is also unsuitable, implying perfect equivalence or total ambiguity, like a “coin toss” between matter and spirit, making the meaning unclear.

Therefore, we chose a special angle: φ = 30° (π/6) , relative to the spiritual axis. This specific geometric orientation is chosen because it represents a context that is dominantly spiritual (cos30° ≈ 0.866) yet retains a significant, non-zero “projection” onto the biological reality (sin30° = 0.5). This perfectly captures the literary nuance, indicating that the fish is primarily a spiritual brother, but its bleeding and suffering remain the empirically real foundations of that spirituality. Thus, our Context Vector can be derived as shown in Equation 7:


$$\begin{array}{l} |C_{conflict}\;\rangle =\;sin(30^{{}^{\circ}})|B\rangle +cos(30^{{}^{\circ}})|S\rangle \;\\ \;=\;0.5|B\rangle +\;0.866|S\rangle \end{array}$$ (7)

### 4.4 Simulation and comparative analysis

When we compare traditional (classical methods) and our quantum method in predicting how a reader integrates a word (target signifier) into a story (narrative structure), the “explanatory gap” between them becomes very clear. We believe that “understanding” corresponds to the probability of the fish's initial state|ψ_*fish*_〉 successfully projecting onto the conflict context state|*C*_*conflict*_〉

If we try to build this model using traditional Classical Probability Theory (Kolmogorovian axioms), we will encounter a significant theoretical limitation. In the traditional classical framework, the “biological” and “spiritual” interpretations of the fish are treated as entirely separate, “disjoint sets”, not like “coherent waves” that can interact. The Law of Total Probability requires us to sum the independent probabilities of each interpretation matching the context. Specifically, assuming equal initial probabilities (prior probabilities) for the biological and spiritual interpretations (both 0.5), we calculate the probability of the biological component aligning with the context (0.25) and add it to the probability of the spiritual component aligning with its respective context (0.75). This way of calculating by treating different parts as independent contributions and simply adding them (component-wise aggregation) results in a total probability of only 0.50 (50%), as calculated in Equation 8:


$$\begin{array}{l} P_{classical}\;=\;(0.5\;\times \;0.25)\;+\;(0.5\;\times \;0.75)\\ \;=\;0.125\;+\;0.375\;=\;0.50 \end{array}$$ (8)

This classical model's result (50% probability) exposes a major flaw: it suggests readers should feel confused or indifferent at this point. It's like a coin toss; meaning is ambiguous because conflicting signals cancel each other out. Mathematically, the classical model is “blind” to phase alignment, implying the fish's bloodiness detracts from its holiness, leaving readers in a semantic “no-man's-land”. However, this prediction completely contradicts our actual experience of reading the novel (a powerful, unitary emotional impact).

However, when we switch to the “Quantum Interference Model”, the outcome changes completely. In quantum mechanics, we don't directly sum probabilities; instead, we first sum “probability amplitudes”. We calculate the “inner product” (overlap) between the word state and the context state. This method preserves phase information, allowing the two dimensions to interact. The total amplitude A is derived by summing the products of the basis components: the biological term (0.707 × 0.5) and the spiritual term (0.707 × 0.866). This summation results in a total amplitude of 0.9658. By applying the “Born Rule” (squaring this amplitude) to calculate the observable probability, we get the result in Equation 9:


$$\begin{array}{l} P_{quantum}\;=\;|A{|}^{2}\;=\;|\left(0.707\;\times \;0.5\right)+\left(0.707\;\times \;0.866\right){|}^{2}\\ \;\approx \;|0.9658{|}^{2}\;\approx \;0.933\;\left(93.3\%\right) \end{array}$$ (9)

The quantum method yields a result (93.3%) that approaches certainty, significantly higher than the classical model's 50% prediction. This significant increase is mathematically attributed to the “Interference Term”. Because the signifier (fish) and the context (narrative) share compatible phase alignment in the Hilbert space (represented by positive real amplitudes), they undergo “Constructive Interference”.

This quantitative result carries profound theoretical implications. It offers rigorous mathematical proof for a phenomenon that literary critics have long intuitively sensed but struggled to formalize. Specifically, it confirms that Hemingway's emphasis on physical detail in the novel does not distract from the spiritual symbolism; instead, it acts as an “amplifier”. This biological reality functions as a carrier wave that reinforces, rather than subtracts from, the spiritual amplitude. This finding aligns with previous work on quantum-like modeling of concepts and interference, showing how context and superposition can produce non-classical probability patterns in conceptual combination and metaphor-like constructions (Aerts et al., 2012; Khrennikov, 2015). By modeling meaning as a wave function, we successfully map the phenomenological experience of insight onto the mathematical convergence of the state vector. This demonstrates that ambiguity, when coherent (or harmonious), is a resource for meaning construction, not just noise to be filtered out.

## 5 The neural substrate of quantum semantic dynamics

For our proposed quantum model to have scientific validity beyond mathematical formalism, it must align with the human brain's material reality. We believe that the wave function characteristics, such as superposition, interference, and collapse, observed in our Hemingway analysis, resemble and potentially describe the “nonlinear” and “oscillatory dynamics” within our cortical networks. This view is consistent with the brain's known complex, dynamic information processing capabilities (Bai et al., 2022).

The cognitive state of Semantic Superposition finds its “biological hardware” in the cortex's oscillatory architecture. While classical linguistics often treats words as static, Libben (2017) proposed “Wave/Particle Duality” for words in the mental lexicon, meaning a word can be both a discrete unit and dynamic potential. Neurophysiologically, this “wave nature” exists through Neural Oscillations. Modern “magnetoencephalography (MEG)” evidence refutes the idea of sequential processing; instead, lexical retrieval triggers rhythmic excitability. Brodbeck et al. (2022) found that during narrative comprehension, the brain maintains multiple linguistic predictions in parallel. This creates a high-entropy reservoir of potentiality, where various semantic networks are weakly activated in low-frequency bands (Theta/Alpha). These low-frequency oscillations are well-suited to maintain and integrate disparate information streams across greater cortical distances, thus physically embodying the coherent quantum state before “measurement”.

In this oscillatory environment, the mathematical mechanism of “Quantum Interference”, represented by the cos(θ) term in our Equation (3), can be directly explained by phase synchronization and desynchronization between different neural assemblies in the brain. The Communication through Coherence (CTC) hypothesis (Fries, 2015) and related research show that effective communication among neuronal groups critically depends on their temporal alignment within specific frequency bands (Vinck et al., 2023; Reyner-Parra and Huguet, 2021). If the “neural population” encoding a narrative context (e.g., The Old Man) oscillates in phase with the population encoding a target signifier (e.g., Fish), their signals summate more effectively at postsynaptic targets and are more likely to cause spiking. This is a physiological analog of constructive interference. Conversely, if their oscillations are out-of-phase or weakly synchronized, it reduces “effective synaptic impact” or diverts signals elsewhere. This mirrors the “destructive interference patterns” seen in our simulation, where conflicting semantic dimensions fail to converge on a unified interpretive state.

So, the transition from uncertainty to clarity, which we call “state vector collapse”, at the cognitive level, can be seen as a rapid reconfiguration of distributed representations in our brain. Surov et al. (2021) proposed a quantum probabilistic model of text perception. Here, superposition and interference of cognitive states concisely explain how sentence structure shapes our understanding of semantic connections, going beyond traditional vector-space and classical probability approaches. At the neural level, we hypothesize this “collapse” is the brain's shift from a more exploratory, distributed processing regime to a more focused state that forms a specific interpretation. This quick shift may be regulated by dynamic changes in neural gain control and selective attention, channeling information flow toward a single, salient interpretation. Neuroimaging work on metaphor and literary reading also shows that complex meaning construction involves interaction between sensorimotor systems and higher-order associative regions. This suggests that meaning integration requires the temporary recruitment and coordination of multiple networks (Mak and Willems, 2021). In our framework, this rapid reconfiguration of distributed activity is like a measurement-like reduction from a space of competing possibilities to one consciously accessible interpretation.

Finally, grounding this theory in biology serves a very pragmatic function: addressing current limitations of Artificial Intelligence. Today's Large Language Models are powerful but rely on static vector embeddings to understand word meaning, struggling with the dynamic, context-dependent nature of meaning. Our quantum-neuro approach suggests future AI architectures must include oscillatory dynamics. By mimicking the brain's phase-dependent processing, we get closer to a computational model that views ambiguity not as noise to filter out, but as a fundamental resource for meaning construction. This framework also provides testable hypotheses for future neurophysiological studies, exploring how manipulating neural phase coherence can directly influence how semantic ambiguities are resolved.

## 6 Pragmatic implications for the design of artificial semantic systems

Our theoretical framework for dynamic meaning construction goes beyond literary analysis or cognitive psychology. It has significant implications for machine intelligence. We've clearly shown how classical probabilistic models fail to capture the superposition and interference in human meaning. This research points AI toward a crucial new direction. Its practical value is in closing the major semantic gap that Generative Artificial Intelligence (GenAI) currently faces. So, moving from classical LLMs to QNLP isn't just an engineering upgrade. It's an essential paradigm shift for machines to process meaning with the same fluidity, ambiguity, and depth as the human mind.

### 6.1 The density matrix as a formalism for dynamic meaning representation

Current generative models, such as LLMs, represent linguistic items as high-dimensional real vectors. They generate text using classical probability to predict the next word. This approach provides impressive fluency. However, it compresses many context-sensitive aspects of meaning into single point embeddings. As quantum-inspired word representation research notes, traditional vector models often “package” various word meanings into one vector. This makes it hard to explicitly show coexisting or competing senses and contextual potentials.

Quantum-inspired language modeling proposes a new method: it treats linguistic units (words, phrases, or sentences) as density matrices within a Hilbert space. This formalism excels at handling both pure states and mixed states. Thus, QLMs and QNLP widely adopt it to model mixtures of semantic subspaces and distributions over meanings (Eisinger et al., 2025). In this framework, a pure state, like a rank-one projection, represents a clearly defined semantic configuration. A mixed state, represented by a density matrix, captures a probabilistic mixture of various semantic components. Here, diagonal entries represent classical probabilities, while off-diagonal structures indicate coherence-like interactions among these semantic components.

Several models have made this idea practical. For example, Zhang et al. (2018) build sentence-level density matrices from word embeddings. They treat each sentence in question-answering tasks as a mixed state over semantic subspaces. Their experiments on TREC-QA and WIKIQA show that these density-matrix representations significantly improve answer selection compared to strong neural baselines. Subsequent work extended this to quantum-like matching using density matrices, combining “state” and “probability” views to unify neural and probabilistic language modeling. This ultimately achieved state-of-the-art results in semantic matching tasks (Zhang et al., 2025).

Building on these developments, a density-matrix-based semantic representation offers compelling reasons to analyze literary meaning. Consider the fish in The Old Man and the Sea: it can be a biological entity (food, animal body) and a deeper spiritual symbol (brother, moral or existential counterpart) simultaneously. In a density-matrix representation, these different interpretations can be seen as distinct basis states or subspaces within a larger semantic Hilbert space. Diagonal entries record the relative importance of each interpretation, while off-diagonal entries denote structured relations or interference-like coupling between them. This allows the system to maintain a mixed, dynamically updatable semantic state, rather than forcing it to a single, context-independent vector.

This view aligns with theoretical and empirical developments in QNLP, which uses density matrices to handle linguistic ambiguity and dynamically update meaning in text contexts. For instance, compositional frameworks like DisCoCat and DisCoCirc systematically integrate density matrices and completely positive maps to represent and evolve meanings within text. In these approaches, density-matrix semantics shows significant advantages over simpler vector semantics, especially in handling inherent ambiguity and facilitating context-dependent semantic updates. Furthermore, some quantum-like sentence representations extend from pure-state density matrices to mixed-state projection operators, which model local word associations. Empirical findings suggest this mixed-state modeling enhances sensitivity to nuanced semantic relations, including negative correlations, across various textual similarity benchmarks (Yu et al., 2024).

From this perspective, the density matrix is more than a complex “box” for word vectors. It provides a powerful formalism, enabling an artificial semantic system to represent coexisting semantic potentials, like conflicting or layered interpretations, as mixed states. Moreover, its off-diagonal structure captures interactions between semantic components, allowing for interference-like effects during composition. Crucially, the density matrix also supports meaning updating through completely positive maps, consistent with quantum-inspired models of discourse and text-level semantics. These features make density matrices a promising mathematical substrate for modeling dynamic, ambiguous, and context-rich meaning. Thus, they naturally become a strong candidate for next-generation semantic architectures aiming to overcome the limitations of static point embeddings.

### 6.2 Quantum interference as a control mechanism for semantic fidelity

Large generative models sometimes “hallucinate”, producing fluent but factually incorrect or contextually inappropriate content. Research shows that hallucination in natural language generation isn't a simple error. It usually stems from a combination of poor training data, flawed generation strategies, and a lack of explicit factual grounding or verification (Liu et al., 2025). From a quantum perspective, hallucination can be seen as the uncontrolled spread of low-quality semantic trajectories within a complex high-dimensional space.

Quantum-like language models propose a new idea: semantics can be treated as quantum states. This means combinations of words and sentences and their matching involve phenomena similar to quantum interference (Fan et al., 2024; Liang et al., 2025). In these models, sentence meaning updates and evolves via unitary-like or completely positive maps. Similarity between sentences or question-answer pairs is calculated by quantum measurements on their representing density matrices. These studies prove that interference-like structures, whether through complex amplitudes, entanglement, or unitary transformations, can precisely express complex semantic relationships and dynamic changes.

Based on these findings, we can imagine a new control method for generative AI: use quantum interference-like mechanisms to actively suppress illogical or factually incorrect content, while strengthening contextually supported content. Specifically, in a quantum-inspired semantic engine, a text might have multiple possible continuations. We can assign amplitudes with relative phases to these possibilities. Continuations that fit the context well and are factually grounded will have aligned phases, leading to constructive interference in their overall amplitude, making them stronger. Conversely, contextually inconsistent or factually unsupported continuations will have misaligned phases, causing destructive interference. This will reduce their impact in the final probability distribution.

Although current density-matrix-based QNLP models are not yet full-scale generative systems, related ideas appear in quantum-like control and information-thermodynamic models, where quantum self-organization is employed to enhance robustness and stability under uncertainty. Quantum-inspired intelligent controllers, for example, utilize quantum and soft-computing mechanisms to extract useful information, manage entropy-like quantities, and improve robustness in complex control tasks (Zrelova et al., 2022). At the same time, recent work analyzing errors and hallucinations in AI from an information-theoretic and “semantic entropy” perspective argues that hallucinations stem from rising semantic entropy in closed probabilistic models and consequently suggests the need for new architectures that actively manage this entropy (Bukalov, 2024).

Combining these strands, one can envision a quantum–neural hybrid architecture. In this proposed architecture, the evolving semantic state of a conversation or narrative would be represented as a (possibly mixed) state in a Hilbert space, often realized as a density matrix over semantic subspaces . Candidate continuations, initially generated in a classical or neural manner, would then be lifted into this semantic space as alternative processes or states. A crucial quantum-inspired control layer would subsequently adjust the phases and amplitudes of these alternatives based on factual grounding signals, discourse coherence metrics, and external verification, effectively engineering patterns of constructive and destructive interference before sampling.

In such a system, hallucination suppression would no longer be purely a *post-hoc* filtering problem; instead, it would become an intrinsic property of the semantic dynamics. While this remains a theoretical proposal, it is notably compatible with existing empirical evidence. This evidence suggests that quantum-like models can capture fine-grained semantic interactions and the dynamic evolution of sentence meaning more effectively than purely classical baselines across various tasks, including question answering, text classification, and semantic similarity (Zhang et al., 2025; Liang et al., 2025; Shi et al., 2024).

### 6.3 Photonic hardware and the physical realization of embodied semantics

When we discuss how intelligent systems are truly realized, especially through embodied and photonic methods, a major issue in current Artificial Intelligence becomes clear. Human understanding and meaning inherently arise from our dynamic physical bodies. Yet, many existing AI models act as if semantics are independent of any physical substrate. Human comprehension involves a body and brain with massively parallel, recurrent dynamics, tightly coupled to their environment. In contrast, mainstream deep learning largely retains the von Neumann separation of memory and processing. This causes significant bandwidth and energy bottlenecks during large-scale matrix operations.These inherent hardware constraints fundamentally shape the kinds of “semantic states” that can be sustained in real time.

Emerging photonic and neuromorphic technologies offer excellent alternatives for semantic processing. For example, neuromorphic photonics leverages light's high speed, large bandwidth, and natural parallelism to simulate distributed neural-like dynamics on photonic integrated circuits (Shastri et al., 2020; Wetzstein et al., 2020). These systems are designed for computations challenging for traditional digital chips, such as ultrafast vector–matrix multiplications and real-time learning under strict energy limits. Recent photonic accelerators have already demonstrated inference for large-scale deep networks, including Transformer models, with near-electronic precision (Ahmed et al., 2025). This shows that photonic hardware can more efficiently execute the tensor operations underlying contextual embeddings and attention. In the quantum realm, integrated optical platforms now realize universal two-qubit processors and multidimensional entanglement on large silicon chips, while single-photon devices run variational algorithms and small quantum neural networks (Maring et al., 2024).

These developments suggest that meaning is not just an abstract vector but a physically realized state within an optical circuit. In a photonic semantic processor, contextual composition could be directly implemented by interferometric networks. These networks, in hardware, perform high-dimensional unitary transformations and tensor products. Wave interference patterns inherently encode compositional structure and constraints. In this setting, understanding a semantic element would correspond to a specific configuration of phases and amplitudes in a multimode optical field, stabilized and updated by the circuit's dynamics. While quantum and quantum-inspired language models already use interference, superposition, and entanglement for semantic correlations at the algorithmic level (Li et al., 2021), photonic and quantum-photonic hardware offer a direct way to embed these representations intrinsically into the physics of computation, instead of emulating them on architectures not designed for such dynamic processes.

From this perspective, an “embodied Artificial Intelligence” doesn't strictly need to mimic biological tissue. Instead, it requires a physical substrate whose native dynamics can inherently sustain rich, overlapping, and rapidly reconfigurable semantic states. Photonic neuromorphic and quantum-photonic processors, with their unique combination of high-dimensional Hilbert spaces, intrinsic interference capabilities, and low-latency parallelism, provide a clear path toward realizing such physically embodied semantics.

### 6.4 Quantum game theory and the strategic negotiation of meaning

If we view semantics as constantly changing states within a complex high-dimensional space, human communication fundamentally transforms into a process where agents strategically prepare, adjust, and interpret these states. Thus, the negotiation of meaning between a speaker and a listener can be seen as a game. Each participant, uncertain of the other's true intentions, chooses their “actions”: how to speak, how to interpret, or how to clarify. While classical game theory effectively analyzes strategic interactions, it assumes players' choices at each step are definite and fixed. Quantum game theory, however, goes further. It allows strategies to be superposed, correlated, and transformed within Hilbert spaces. This significantly broadens the decision-making possibilities and fundamentally alters the structure of game equilibria.

Quantum games show that even without the advanced phenomenon of entanglement, merely utilizing superposition (considering multiple possibilities simultaneously) and coherence (maintaining relationships between these possibilities) can yield significant strategic advantages. For example, coherent mixtures of strategies, represented by density matrices, have been shown to outperform any classical randomized strategy in certain non-zero-sum settings (Sanz-Martín et al., 2025). More broadly, quantum strategic formalisms use positive semidefinite operators, subject to linear constraints, to model each player's behavior. In this model, Nash equilibria correspond to fixed points in these operator spaces. This sophisticated mathematical framework is increasingly applied in Artificial Intelligence fields like learning-in-games and multi-agent reinforcement learning, where quantum common-interest games and quantum replicator dynamics have been proposed as frameworks for decentralized optimization over density-matrix strategy spaces (Lin et al., 2023).

From a semantic perspective, these theoretical insights suggest that ambiguity and the superposition of intentions should not be treated as noise, but rather as valuable communicative resources. For instance, a communicating AI might formulate an utterance that allows for several different, even partially contradictory, interpretations. It would then use contextual feedback from the interlocutor to gradually “probe” and refine the shared semantic state. Quantum-inspired language and multimodal models already leverage superposition and entanglement to integrate information and capture complex correlations between linguistic units that are difficult for classical methods to discover. Quantum game theory extends this logic to the interaction itself: agents can be modeled as maintaining superposed stance or intent states, dynamically adjusting them through iterative exchanges. They seek an equilibrium that not only maximizes material payoffs but also promotes alignment of perspectives and affective impact (Capraro et al., 2024).

For artificial semantic systems, this new paradigm implies a fundamental design shift. Future “quantum-aware” AI agents should not view ambiguity, irony, or layered metaphor as “flaws” to be eliminated. Instead, they should deliberately maintain multiple potentially compatible or incompatible interpretive hypotheses. They would then use game-theoretic feedback to update a density-matrix-like representation of their communicative state. Quantum and quantum-inspired game frameworks already point toward such interaction models in which the core computational object is not a single best response, but a structured distribution over possible moves and meanings. Incorporating these ideas into AI architectures would fundamentally alter the direction of system design, making AI focus on strategically understanding meaning through negotiation. This will pave a new path for AI, moving it from current narrowly task-optimized dialogues toward understanding content with richer cultural and pragmatic significance.

## 7 Conclusion

This study primarily addresses a core paradox in cognitive science: how the human brain, with its finite structure and resources, manages to generate and comprehend such infinite, fluid, and often contradictory meanings. We argue that most current computational methods, by treating words as mere static “points” or “particles”, are too rigid to capture this dynamic nature of meaning. Therefore, we propose a new approach, combining the concept of “superposition” from classical cognitive science with Quantum Probability Theory, to redefine our mental lexicon. Under this new framework, a word is no longer a collection of fixed definitions in the mind but a dynamic system exhibiting wave-particle duality. A word, in its resting state, exists as a “wave” full of various possibilities; it only “collapses” into a specific, particle-like meaning when “measured” by a particular story or context.

Our computer simulation of Hemingway's The Old Man and the Sea strongly validated this “wave-particle duality” perspective. We mathematically modeled the tension between realism and spiritual symbolism in the novel. The results showed that quantum probability principles, specifically superposition and constructive interference, effectively explain and quantify phenomena that classical probability dismisses as “noise”. For instance, a classical mixture model predicted a 50% confusion probability when faced with conflicting semantic cues, but our quantum model accurately predicted a 93.3% probability of coherent meaning emergence. This result provides rigorous mathematical validation for the intuition that context doesn't just filter meaning; it actively adjusts the brain's measurement basis, allowing seemingly contradictory semantic features to resonate and create new understanding instead of canceling each other out. Crucially, this mathematical abstraction is rooted in biological reality: the isomorphism between the quantum phase angle and neural phase synchronization suggests that the wave function is not a vague metaphor, but a functional description of the brain's electromagnetic field dynamics.

While this framework offers a solid theoretical foundation, we must acknowledge its current limitations, which also highlight future research directions. First, our computer simulation was performed in a relatively simple 2-dimensional Hilbert space. While sufficient for demonstrating the interference effect in a binary metaphor, real-world semantic processing involves thousands of interacting semantic features within a far more complex, hyper-dimensional space. Future research will need to employ Tensor Network states to model these higher-order interactions without incurring exponential computational costs. Second, we must be cautious regarding the ontological interpretation of the “Quantum Brain”. We are not claiming the brain is a quantum computer maintaining sub-atomic coherence at room temperature. Instead, we advocate for “Quantum-Like Cognition” that the brain, as a macroscopic biological system, has evolved to process information using non-classical probability rules because these are evolutionarily more efficient for handling uncertainty.

Despite these constraints, our model's practical implications extend beyond literary theory, charting a transformative path for both psychology and Artificial Intelligence. For cognitive science, this model suggests a new experimental agenda: using “Priming-EEG” paradigms to detect specific oscillatory signatures of destructive vs. constructive semantic interference during meaning comprehension. For Artificial Intelligence, our findings identify the “static vector bottleneck” as the fundamental cause of hallucinations in current Large Language Models (like GPT). Furthermore, the Quantum Game Theory principles discussed earlier provide a blueprint for next-generation AI agents. These agents could utilize “Strategic Ambiguity” to better understand and negotiate meaning in complex social interactions. We believe that building truly intelligent AI, especially systems capable of grasping the deep meaning of metaphors, not just their word frequencies, requires a shift to Quantum Natural Language Processing (QNLP) architectures and Photonic Hardware that uses Density Matrices to model semantic entanglement.

Ultimately, just as meaning is more than a mathematical vector, the novel's “Big Fish” is more than a simple creature. It represents a dynamic event: the sudden convergence of infinite possibilities into a single moment of understanding. Embracing the quantum nature of this process allows us to connect precise mathematics with the profound depth of human experience, bringing us closer to uncovering the physics of the mind.
